# The Geriatric G8 Score Is Associated with Survival Outcomes in Older Patients with Advanced Prostate Cancer in the ADHERE Prospective Study of the Meet-URO Network

**DOI:** 10.3390/curroncol29100612

**Published:** 2022-10-14

**Authors:** Giuseppe Luigi Banna, Umberto Basso, Emilio Francesco Giunta, Lucia Fratino, Sara Elena Rebuzzi, Sebastiano Buti, Marco Maruzzo, Ugo De Giorgi, Veronica Murianni, Marika Cinausero, Helga Lipari, Teresa Gamba, Orazio Caffo, Davide Bimbatti, Arianna Dri, Alessandra Mosca, Paola Ermacora, Francesca Vignani, Aichi Msaki, Barbara Bonifacio, Valentina Lombardo, Vincenza Conteduca, Giuseppe Fornarini, Pasquale Rescigno

**Affiliations:** 1 Portsmouth Hospitals University NHS Trust, Portsmouth SO16 6YD, UK; 2 Medical Oncology 1 Unit, Department of Oncology, Istituto Oncologico Veneto IOV IRCCS, 35128 Padova, Italy; 3 Candiolo Cancer Institute, FPO-IRCCS, 10060 Candiolo, Italy; 4 Department of Medical Oncology, Centro di Riferimento Oncologico di Aviano CRO-IRCCS, 33081 Aviano, Italy; 5 Medical Oncology Unit, Ospedale San Paolo, 17100 Savona, Italy; 6 Department of Internal Medicine and Medical Specialties (Di.M.I.), University of Genova, 16132 Genova, Italy; 7 Medical Oncology Unit, University Hospital of Parma, 43126 Parma, Italy; 8 Department of Medical Oncology, IRCCS Istituto Romagnolo per lo Studio dei Tumori (IRST) “Dino Amadori”, 47014 Meldola, Italy; 9 Medical Oncology Unit 1, IRCCS Ospedale Policlinico San Martino, 16132 Genova, Italy; 10 Department of Oncology, ASUFC Santa Maria Della Misericordia, 33100 Udine, Italy; 11 Division of Medical Oncology, Cannizzaro Hospital, 95126 Catania, Italy; 12 Medical Oncology, Mauriziano Hospital, 10128 Turin, Italy; 13 Department of Medical Oncology, Santa Chiara Hospital, 38122 Trento, Italy; 14 Department of Medicine, University of Udine, 33100 Udine, Italy; 15 Department of Medical and Surgical Sciences, Unit of Medical Oncology and Biomolecular Therapy, University of Foggia, Policlinico Riuniti, 71122 Foggia, Italy

**Keywords:** G8 score, geriatric assessment, adherence to treatment, prostate cancer

## Abstract

**Introduction:** Androgen receptor pathway inhibitors (ARPIs) have been increasingly offered to older patients with prostate cancer (PC). However, prognostic factors relevant to their outcome with ARPIs are still little investigated. **Methods and Materials:** The Meet-URO network ADHERE was a prospective multicentre observational cohort study evaluating and monitoring adherence to ARPIs metastatic castrate-resistant PC (mCRPC) patients aged ≥70. Cox regression univariable and multivariable analyses for radiographic progression-free (rPFS) and overall survival (OS) were performed. Unsupervised median values and literature-based thresholds where available were used as cut-offs for quantitative variables. **Results:** Overall, 234 patients were enrolled with a median age of 78 years (73–82); 86 were treated with abiraterone (ABI) and 148 with enzalutamide (ENZ). With a median follow-up of 15.4 months (mo.), the median rPFS was 26.0 mo. (95% CI, 22.8–29.3) and OS 48.8 mo. (95% CI, 36.8–60.8). At the MVA, independent prognostic factors for both worse rPFS and OS were Geriatric G8 assessment ≤ 14 (*p* < 0.001 and *p* = 0.004) and PSA decline ≥50% (*p* < 0.001 for both); time to castration resistance ≥ 31 mo. and setting of treatment (i.e., post-ABI/ENZ) for rPFS only (*p* < 0.001 and *p* = 0.01, respectively); age ≥78 years for OS only (*p* = 0.008). **Conclusions:** Baseline G8 screening is recommended for mCRPC patients aged ≥70 to optimise ARPIs in vulnerable individuals, including early introduction of palliative care.

## 1. Introduction

Prostate cancer is the fourth most common cancer worldwide [1]. Age-standardized incidence and mortality are 68 and 10 per 100,000, respectively, in more developed regions [2]. The average age at which prostate cancer (PC) is diagnosed in western populations is 66 years old. However, at the time of diagnosis, 60% of patients are 65 years or older, and by 2040, this percentage will rise to 70% [3]. Nearly 70% of PC deaths occur in men aged ≥ 75 [1]. While the overall mortality rate is predicted to remain steady, the number of men aged 70 and older who die from prostate cancer will nearly double by 2040 [1]. Furthermore, the median age of men who develop the metastatic disease is considerably older, and the median age of those who die due to prostate cancer is eighty years [4]. Treatment costs for older men with early and late prostate cancer are already high and projected to rise in the following decades [3]. Androgen receptor pathway inhibitors (ARPIs) represent the standard of care for advanced PC (APC). ARPIs are often preferred to intravenous chemotherapy for older patients with metastatic castrate-resistant PC (mCRPC) due to their more favourable toxicity profile and convenient administration [5]. However, treatment choice should not be based on patients’ age but on the overall evaluation of the patients’ health status. The gold standard for health status evaluation of older patients is the Comprehensive Geriatric Assessment (CGA), although it is time-consuming and requires a specialist assessment [6]. The Geriatric G8 (G8) score assesses, with eight questions, the patient’s food intake, weight loss, body mass index, mobility, neuropsychological problems, polypharmacy, self-perceived health status and age. An abnormal G8 score (>14 on a scale from 0 to 17) was strongly associated with mortality in almost a thousand cancer male patients aged ≥ 70 [7]. We have previously reported that the G8 score is an associated factor with adherence to ARPIs in older mCRPC patients [8,9,10]. In the current analysis, we investigated the clinical baseline and on-ARPI variables associated with radiographic progression-free survival (rPFS) and overall survival (OS).

## 2. Methods

The ADHERE was a Meet-URO network prospective multicentre observational study monitoring adherence to abiraterone (ABI) or enzalutamide (ENZ) in patients with mCRPC aged ≥ 70. To assess the prognostic factors on rPFS and OS, a Cox regression univariable analysis (UVA) was performed, including the following clinical characteristics: baseline prior ARPI start, Gleason score (≥8 vs. <8), surgery on primary (yes vs. no), time to CR (≥31 vs. <31 months [mo.]), baseline at ARPI start, age (≥78 vs. <78), sites of metastases (lymph nodes only vs. bone vs. visceral), setting of therapy (pre- vs. post-chemotherapy vs. post-ABI/ENZ), steroid use (yes vs. no), Charlson comorbidity score (≥10 vs. <10), G8 (≤14 vs. >14), IADL (≤6 vs. >6), number of concomitant therapies (≥3 vs. <3), caregiver presence (yes vs. no); on-ARPI characteristics, type of therapy (ABI vs. ENZ), PSA decline by 50% (PSA50) (yes vs. no), grade 1/2 toxicity (yes vs. no), grade 3/4 toxicity (yes vs. no). The rPFS was defined as the time from ARPI start to date of disease progression on imaging as per RECIST 1.1, or death from any cause, whichever occurred first. OS was calculated from the ARPI start date until death or the last follow-up. Unsupervised median values were used as cut-offs for quantitative variables alongside the literature-reported values of 9 for Charlson comorbidity score [11], <12 months for time to castration resistance (CR) [12] and ≥75 for age. Cox regression multivariable analysis (MVA) was performed for OS and PFS of clinical variables with a *p*-value < 0.05 at the UVA. When both median and literature-reported cut-off values of quantitative variables were significant, the one with the lowest *p*-value was carried on in the MVA. Kaplan–Meier curves were used for time-to-event analyses. The analysis was performed using the statistical software SigmaPlot v12.5 (Systat Sotware, Inc., Dusseldorf, Germany).

## 3. Results

The characteristics of patients in the overall cohort and the ARPI-relative ones are summarized in Supplementary Table S1. Among the 234 enrolled patients, the median age of 78 years (73–82); 86 were treated with ABI and 148 with ENZ. With a median follow-up of 15.4 months (mo.) (95% confidence interval [CI], 12.1–18.7), the median rPFS was 26.0 mo. (95% CI, 22.8–29.3) and OS 48.8 mo. (95% CI, 36.8–60.8).

At UVA, age ≥ 78 or ≥75 was associated with worse OS (*p* = 0.004 or *p* = 0.014) but not shorter rPFS (*p* = 0.077 or *p* = 0.090); time to CR < 31 or <12 mo. and Charlson score ≥ 9 were associated with worse rPFS (*p* = 0.002 or *p* = 0.007, *p* = 0.034) but not shorter OS (*p* = 0.111 or *p* = 0.168, *p* = 0.361); while G8 ≤ 14 and lack of biochemical response were both associated with worse OS (*p* < 0.001 for both) and rPFS (*p* = 0.032, *p* < 0.001) (Table 1). Interestingly, presence of a caregiver and treatment with ENZ (vs. ABI) were associated with shorter rPFS (*p* = 0.047, *p* = 0.042) (Table 1). 

**Figure 1:**
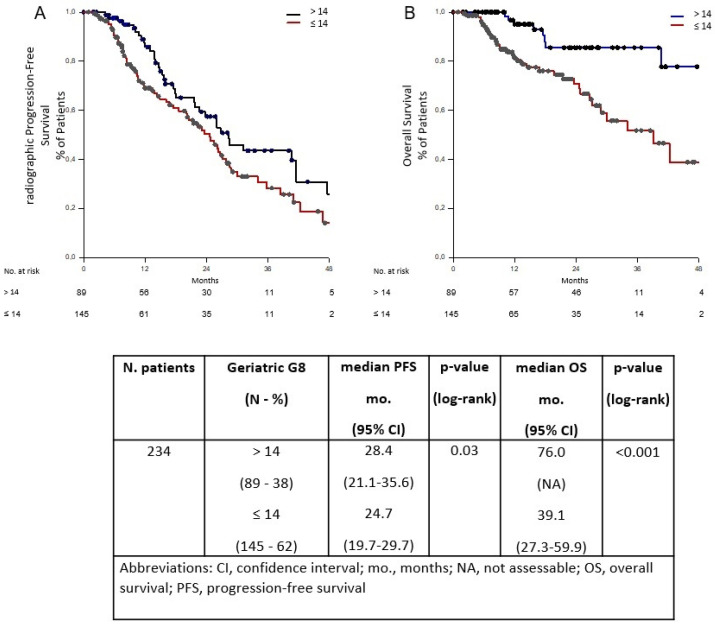
Radiographic progression-free survival (rPFS) (**A**) and overall survival (OS) (**B**) based on Geriatric G8 with cut-off of 14. Abbreviations: CI, confidence interval; mo., months; NA, not assessable; OS, overall survival; rPFS, radiographic progression-free survival.

At MVA, independent prognostic factors for both worse rPFS and OS were G8 ≤ 14 (*p* < 0.001 and *p* = 0.004) and PSA decline ≥ 50% (*p* < 0.001 for both). Time to CR ≥ 31 mo. and ARPI setting (i.e., post-ABI/ENZ) were associated factors with the rPFS only (*p* < 0.001 and *p* = 0.01, respectively), whilst age ≥ 78 years with the OS only (*p* = 0.008) (Table 2). Eighty-nine (38%) patients presented with a G8 > 14, while 145 (62%) had a score ≤ 14 (Table 1 and Supplementary Figure S1). The median rPFS for patients with G8 ≤ 14 vs. >14 was 24.7 mo. (95% confidence interval [CI], 19.7–29.7) vs. 28.4 mo. (95% CI, 21.1–35.6) (*p* = 0.03). There was also a statistically significant difference in OS between those two groups (39.1 mo. [95% CI, 27.3–59.9] vs. 76.0 mo. [95% CI, not assessable], *p* < 0.001) as presented in Figure 1.

## 4. Discussion

Frailty is a complex interplay of illness and health, personal attitudes, reliance on others, and resources [13]. Therefore, CGA, which explores comorbidities, mental health and cognitive status, functional status, nutrition, social status and support, polypharmacy, and geriatric syndromes, represents the perfect tool to assess such a multifaceted condition as frailty in older cancer patients [14,15]. 

The aim of CGA is to identify patients who need optimization of medical treatments, thus improving their prognosis, by restoring autonomy or, where possible, supporting its loss to ameliorate overall patients’ quality of life [16]. However, the CGA is time-consuming, implies the presence of geriatric team, and has too many elements that need to be tested, making its use unsuitable for many oncology centers [17].

International medical societies, like the European Organization for Research and Treatment of Cancer (EORTC), European Association of Urology (EAU) and International Society of Geriatric Oncology (SIOG), all recommend screening cancer patients aged ≥ 70 with the G8 since this was proven to identify those requiring a more complex CGA [7]. Nevertheless, as emerged in recent surveys, up to half of the clinicians use merely the performance status (PS) by Karnofsky (KPS) or Eastern Cooperative Oncology Group (ECOG PS) scores to assess patient’s frailty and select older patients for chemotherapy [18,19,20]. Unfortunately, these scores consider physical functioning only, neglecting psychosocial, nutritional and cognitive aspects, which are crucial to evaluating patient’s frailty status [21].

In addition, nearly 40% of the physicians admit to ignoring the G8 scale, and only 50% declare to use it in clinical practice, without apparent regional differences [22].

Moreover, recommendations for using the G8 score from international guidelines were extended to older PC patients without ad hoc prospective studies. By the present study, which focused on older patients with mCRPC treated with ARPIs, we could confirm the relevance of geriatric assessment and G8 as a reliable screening tool. In this setting, G8 can select patients who deserve comprehensive geriatric assessment (CGA) to identify frail or vulnerable patients. As suggested by the International Society of Geriatric Oncology (SIOG) [3], best supportive care (BSC) remains the preferred option for the formers. Conversely, treatment for vulnerable patients carrying reversible clinical conditions should be considered, including the early introduction of palliative care.

While PSA responses have been invariably associated with PFS and OS on hormonal treatments, without satisfying Prentice’s criteria for surrogacy [23], to the best of our knowledge, this is the first prospective study aiming to evaluate the G8 screening in mCRPC and showing its correlation with rPFS and OS. In the ADHERE study, we could not demonstrate an association between a reduced adherence to ARPIs and rPFS or OS, although the G8 was significantly linked to treatment adherence [10]. Therefore, we reckon that the reduced OS and rPFS of patients with a G8 score > 14 likely mirror the general health status as demonstrated in other cancer patients. 

The results presented here, however, do not derive from a pre-planned analysis and lack internal or external validation. Furthermore, a higher number of events would have improved the accuracy of the UVA and MVA estimates, despite the median follow-up being adequate in metastatic castrate-resistant prostate setting. These represent the main limitations of our work.

## 5. Conclusions

Our analysis shows that, in a prospective observational study on older mCRPC patients treated with ARPIs, G8 screening is a baseline prognostic factor for rPFS and OS. Therefore, we envision that G8 will become a baseline screening tool for treatment decisions, as already recommended by international guidelines. 

## Figures and Tables

**Table 1:** Cox regression univariable analysis of clinical baseline and on-treatment factors.

Variable	No. (%)	OS HR (95% CI)	*p*-Value	rPFS HR (95% CI)	*p*-Value
Age ^a^, median, years					
<78	121 (52)	1.00 (ref)		1.00 (ref)	
≥78	113 (48)	2.51 (1.35–4.6)	**0.004**	1.42 (0.96–2.10)	0.077
<75	84 (36)	1.00 (ref)		1.00 (ref)	
≥75	150 (64)	2.27 (1.77–4.39)	**0.014**	1.41 (0.95–2.11)	0.090
Gleason score, median					
<8	78 (36)	1.00 (ref)		1.00 (ref)	
≥8	136 (64)	1.27 (0.68–2.36)	0.449	1.60 (1.04–2.46)	**0.032**
Surgery at diagnosis					
No	146 (62)	1.00 (ref)		1.00 (ref)	
Yes	88 (38)	0.55 (0.30–1.01)	0.055	0.64 (0.43–0.95)	**0.025**
Time to CR, mo, median					
≥31	118 (50)	1.00 (ref)		1.00 (ref)	
<31	116 (50)	1.60 (0.90–2.85)	0.111	1.84 (1.25–2.70)	**0.002**
≥12	183 (78)	1.00 (ref)		1.00 (ref)	
<12	51 (22)	1.57 (0.83–3.00)	0.168	1.76 (1.16–2.66)	**0.007**
Sites of metastases ^a^					
Bone (non-visceral)	163 (70)	1.00 (ref)		1.00 (ref)	
Lymph nodes (only)	49 (21)	0.00 (NA)	0.997	0.31 (0.16–0.62)	**<0.001**
Visceral	22 (9)	20.7 (NA)	0.428	0.80 (0.43–1.49)	0.488
Setting of therapy					
Post-chemotherapy	57 (24)	1.00 (ref)		1.00 (ref)	
Pre-chemotherapy	162 (69)	0.78 (0.41–1.50)	0.350	0.79 (0.51–1.23)	0.300
Post-Abi/Enza	15 (6)	2.06 (0.45–9.41)	0.451	3.68 (1.57–8.66)	**0.003**
Steroid use ^b^					
No	134 (57)	1.00 (ref)		1.00 (ref)	
Yes	100 (43)	1.02 (0.57–1.84)	0.935	0.84 (0.57–1.24)	0.378
Charlson score, median					
≥10	175 (75)	1.00 (ref)		1.00 (ref)	
<10	59 (25)	0.99 (0.52–1.89)	0.977	0.96 (0.62–1.47)	0.846
≥9	190 (81)	1.00 (ref)		1.00 (ref)	
<9	44 (19)	0.70 (0.32–1.51)	0.361	0.55 (0.32–0.96)	**0.034**
Geriatric G8, median					
>14	145 (62)	1.00 (ref)		1.00 (ref)	
≤14	89 (38)	3.58 (1.72–7.49)	**<0.001**	1.55 (1.04–2.31)	**0.032**
IADL, median					
>6	121 (52)	1.00 (ref)		1.00 (ref)	
≤6	113 (48)	1.60 (0.88–2.91)	0.123	1.11 (0.76–1.63)	0.576
Concomitant therapies, no.					
≥3	132 (56)	1.00 (ref)		1.00 (ref)	
<3	102 (44)	1.07 (0.60–1.93)	0.815	0.89 (0.61–1.31)	0.550
Caregiver					
Yes	190 (81)	1.00 (ref)		1.00 (ref)	
No	44 (19)	0.46 (0.18–1.16)	0.098	0.57 (0.32–0.99)	**0.047**
Treatment					
Enza	148 (63)	1.00 (ref)		1.00 (ref)	
Abi	86 (37)	0.74 (0.40–1.36)	0.329	0.65 (0.43–0.99)	**0.042**
PSA50					
No	65 (28)	1.00 (ref)		1.00 (ref)	
Yes	164 (71)	0.18 (0.10–0.32)	**<0.001**	0.25 (0.17–0.37)	**<0.001**
Toxicity, G1/G2					
No	100 (43)	1.00 (ref)		1.00 (ref)	
Yes	134 (57)	1.58 (0.83–2.99)	0.164	1.07 (0.72–1.59)	0.732
Toxicity, G3/G4					
No	222 (95)	1.00 (ref)		1.00 (ref)	
Yes	12 (5)	2.53 (0.90–7.08)	0.077	1.88 (0.87–4.07)	0.107

^a^ At the time of initiation of treatment. ^b^ During the whole treatment. Abi, abiraterone; CI, confidence intervals; CR, castration resistance; Enza, enzalutamide; FU, follow-up; mo., months; G, grade; NA, not assessable; No., number; OS, overall survival; PSA50, decline in the PSA ≥ 50%; ref, reference; rPFS, radiographic progression-free survival; Tx, treatment. Statistically significant values in bold (*p* < 0.005).

**Table 2:** Cox regression multivariable analysis of clinical baseline and on-treatment prognostic factorsa.

Variable	OS HR (95% CI)	*p*-Value	rPFS HR (95% CI)	*p*-Value
Age ^a^, median, years			-	**-**
<78	1.00 (ref)	**0.008**		
≥78	2.47 (1.27–4.79)			
Geriatric G8, median				
>14	1.00 (ref)		1.00 (ref)	
≤14	3.10 (1.43–6.74)	**0.004**	2.39 (1.46–3.91)	**<0.001**
PSA50				
No	1.00 (ref)		1.00 (ref)	
Yes	0.14 (0.07–0.25)	**<0.001**	0.29 (0.18–0.46)	**<0.001**
Gleason score, median	**-**	**-**		
≥8			1.00 (ref)	
<8			0.82 (0.49–1.38)	0.457
Surgery at diagnosis	**-**	**-**		
Yes			1.00 (ref)	
No			1.38 (0.87–2.21)	0.173
Time to CR, mo, median	**-**	**-**		
<31			1.00 (ref)	
≥31			2.30 (1.46–3.64)	**<0.001**
Sites of metastases ^a^	**-**	**-**		
Bone (non-visceral)			1.00 (ref)	
Lymph nodes (only)			0.51 (0.23–1.11)	0.090
Visceral			0.90 (0.46–1.76)	0.748
Setting of therapy	**-**	**-**		
Post-chemotherapy			1.00 (ref)	
Pre-chemotherapy			0.69 (0.42–1.15)	0.157
Post-Abi/Enza			4.31 (1.42–13.04)	**0.010**
Charlson score, median	**-**	**-**		
≥9			1.00 (ref)	
<9			0.61 (0.34–1.11)	0.109
Caregiver	**-**	**-**		
Yes			1.00 (ref)	
No			0.69 (0.36–1.31)	0.251
Treatment	**-**	**-**		
Enza			1.00 (ref)	
Abi			0.75 (0.46–1.19)	0.221

Abbreviations: Abi, abiraterone; BM, bone metastases (non-visceral); ChT, chemotherapy; CI, confidence intervals; CR, castration resistance; Enza, enzalutamide; HR, hazard ratio; LN, lymphnodes; No. Number; OS, overall survival; PSA50, decline in the PSA ≥ 50%; ref, reference; rPFS, radiographic progression-free survival; Tx, treatment. ^a^ Only for variables with a *p*-value < 0.05 at univariable analysis. Statistically significant values in bold (*p* < 0.005).

## Data Availability

The datasets generated and analysed during the current study are not publicly available as they are part of the confidential medical record but are available from the corresponding author on reasonable request.

## Supplementary Materials

The following supporting information can be downloaded at: https://www.mdpi.com/article/10.3390/curroncol29100612/s1, Table S1: Patient characteristics in the whole ADHERE study cohort and by ARPI. Figure S1: Histogram of distribution of patients based on Geriatric G8 score.
