# Supplementary material for: The Geriatric G8 Score Is Associated with Survival Outcomes in Older Patients with Advanced Prostate Cancer in the ADHERE Prospective Study of the Meet-URO Network
Source: Curr Oncol. 2022 Oct 14;29(10):7745–53. doi: 10.3390/curroncol29100612 (PMC9600362; doi:10.3390/curroncol29100612)
Supplement: Supplementary file 1 [file curroncol-29-00612-s001.zip › curroncol-1910478-supplementary.pdf]

Title: The geriatric G8 score is associated with survival outcomes in older patients with advanced prostate cancer in the ADHERE prospective study of the Meet-URO network.

Supplementary Table S1. Patient characteristics in the whole ADHERE study cohort and by ARPI

Characteristic	Abiraterone Cohort (*n* = 86) Value No. (%) [IQR, *95% CI*]	Enzalutamide Cohort (*n* = 148) Value No. (%) [IQR, *95% CI*]	All patients (*n* = 234) Value No. (%) [IQR, *95% CI*]	P Value
Age[a], median, years	78 [73-82]	77.5 [73-82]	78 [73-82]	0.50
Gleason score, median	8 [7-9]	8 [7-9]	8 [7-9]	0.194
Surgery at diagnosis	38 (44)	50 (34)	88 (38)	0.149
Time to CR, mo, median	39.3 [20.5-63.4]	30.1 [11.8-57.1]	31.4 [14.7-58.6]	0.072
Sites of metastates[a]				
Lymph nodes (only)	20 (23)	29 (20)	49 (21)	0.480
Bone (non-visceral)	56 (65)	107 (72)	163 (70)	
Visceral	10 (12)	12 (8)	22 (9)	
PSA at Tx start, median	11.8 [3.1-29.9]	14.3 [5.4-35.9]	14.0 [4.5-33.9]	0.325
Setting of therapy				
Pre-chemotherapy	61 (71)	101 (68)	162 (69)	0.222
Post-chemotherapy	17 (20)	40 (27)	57 (24)	
Post-Abi/Enza	8 (9)	7 (5)	15 (6)	
Steroid use[b] (yes)	86 (100)	14 (9)	100 (43)	**<0.001**
Charlson score, median	10 [8-11]	10 [10-12]	10 [9-11]	**0.028**
Geriatric G8, median	14 [12-16]	13 [12-15]	14 [12-15]	0.081
IADL, median	6 [5-8]	6 [5-8]	6 [5-8]	0.639

Concomitant therapies, no.	3 [1-5]	3 [1-5]	3 [1-5]	0.671
Caregiver (yes)	71 (83)	119 (80)	190 (81)	0.816

[a]At the time of initiation of treatment.

[b]During the whole treatment.

Abi, abiraterone; ARPI, androgen receptor pathway inhibitors; CI, confidence intervals; CR, castration resistance; Δ, difference between the PSA basal value and the best response value; Enza, enzalutamide; mo., months; G, grade; IQR, interquartile range; Tx, treatment; yr, year.

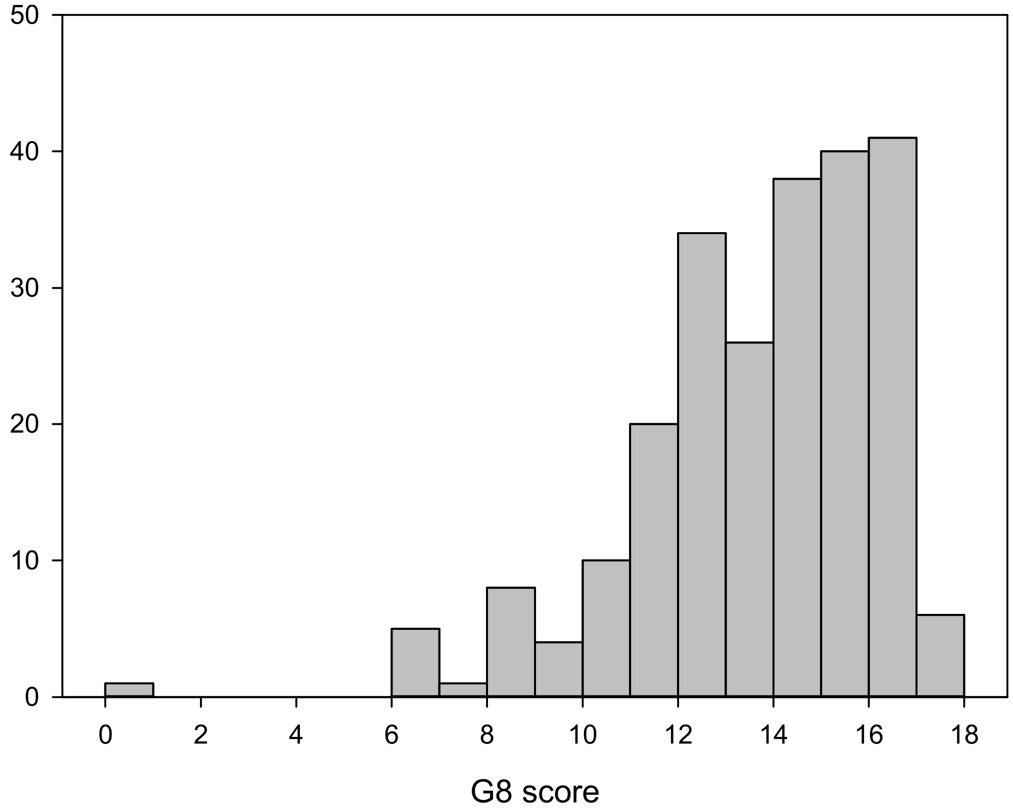

Supplementary Figure S1. Histogram of distribution of patients based on Geriatric G8 score
